# Development of a web-based tool for the assessment of health and economic outcomes of the European Innovation Partnership on Active and Healthy Ageing (EIP on AHA)

**DOI:** 10.1186/1472-6947-15-S3-S4

**Published:** 2015-09-04

**Authors:** Christian EH Boehler, Gimon de Graaf, Lotte Steuten, Yaling Yang, Fabienne Abadie

**Affiliations:** 1 Information Society Unit, European Commission Directorate General Joint Research Centre (DG-JRC) Institute for Prospective Technological Studies (IPTS), Edificio Expo, Calle Inca Garcilaso 3, 41092 Seville, Spain; 2 Panaxea B.V., Hengelosestraat 221, 7521 AC Enschede, The Netherlands; 3 Hutchinson Institute for Cancer Outcomes Research, Fred Hutchinson Cancer Research Center, 1110 Fairview Avenue North, Seattle, WA 98109, USA; 4 Nuffield Department of Primary Care Health Science, Oxford University, New Radcliffe House, Walton Street, Oxford, OX2 6NW, United Kingdom

**Keywords:** decision analytic modelling, health and economic outcomes, active and healthy ageing, European Innovation Partnership, web-based tool

## Abstract

**Background:**

The European Innovation Partnership on Active and Healthy Ageing (EIP on AHA) is a European Commission led policy initiative to address the challenges of demographic change in Europe. For monitoring the health and economic impact of the social and technological innovations carried out by more than 500 stakeholder's groups ('commitments') participating in the EIP on AHA, a generic and flexible web-based monitoring and assessment tool is currently being developed.

**Aim:**

This paper describes the approach for developing and implementing this web-based tool, its main characteristics and capability to provide specific outcomes that are of value to the developers of an intervention, as well as a series of case studies planned before wider rollout.

**Methods:**

The tool builds up from a variety of surrogate endpoints commonly used across the diverse set of EIP on AHA commitments in order to estimate health and economic outcomes in terms of incremental changes in quality adjusted life years (QALYs) as well as health and social care utilisation. A highly adaptable Markov model with initially three mutually exclusive health states ('baseline health', 'deteriorated health' and 'death') provides the basis for the tool which draws from an extensive database of epidemiological, economic and effectiveness data; and also allows further customisation through remote data entry enabling more accurate and context specific estimation of intervention impact. Both probabilistic sensitivity analysis and deterministic scenario analysis allow assessing the impact of parameter uncertainty on intervention outcomes. A set of case studies, ranging from the pre-market assessment of early healthcare technologies to the retrospective analysis of established care pathways, will be carried out before public rollout, which is envisaged end 2015.

**Conclusion:**

Monitoring the activities carried out within the EIP on AHA requires an approach that is both flexible and consistent in the way health and economic impact is estimated across interventions and commitments. The added value for users of the MAFEIP-tool is its ability to provide an early assessment of the likelihood that interventions in their current design will achieve the anticipated impact, and also to identify what drives interventions' effectiveness or efficiency to guide further design, development or evaluation.

## Background

European Union Member States are facing a major societal challenge resulting from demographic change, as ageing populations are more likely to suffer from chronic diseases and have higher demands for health and social care services [1,2]. This demographic trend is putting at risk the sustainability of health and social care systems, and increasing demand for care will have to be met with ever more limited resources as health and care budgets are already under strong pressure [2]. In this context, prevention, care re-organisation and the use of ICT to enhance the efficiency of care delivery are becoming essential for health and social care systems to face the ever increasing demand adequately [3]. The European Innovation Partnership on Active and Healthy Ageing (EIP on AHA) launched by the European Commission in 2011 under the Innovation Union policy initiative is an attempt to address the challenges of ageing in Europe [4]. It provides a platform for stakeholders to join forces, learn from each other and implement interventions that will help improve the quality of life and health status of European citizens and the sustainability of health and care systems, while contributing to economic growth in Europe. Besides these three objectives, also called the "Triple Win", the ultimate goal of the EIP on AHA is to increase the healthy lifespan of European citizens by two healthy life years (HLY) by 2020 [4].

**Figure 1:**
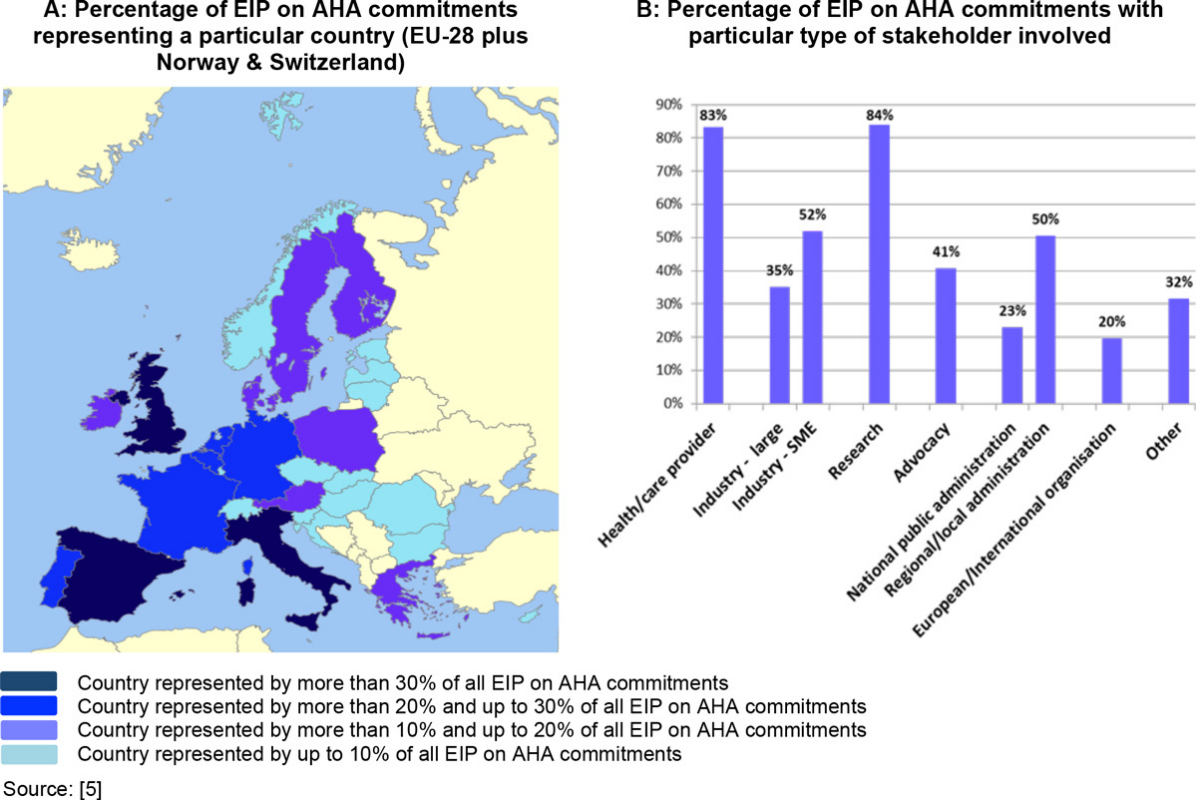
**Stakeholders and countries represented in the EIP on AHA**.

In order to monitor the progress of the EIP on AHA initiative towards these objectives, the 'Monitoring and Assessment Framework for the EIP on AHA (MAFEIP) project' was launched jointly by the European Commissions' (EC) Joint Research Centre, Institute for Prospective Technological Studies (JRC IPTS), the Directorate General for Communications Networks, Content and Technology (DG CNECT), and the Directorate General for Health and Food Safety (DG SANCO) [4]. The aim was to develop and implement a framework that can help estimating the health and economic outcomes of a large variety of social and technological innovations in the health and care sector targeting active and healthy ageing. These innovations are being developed and implemented by a total of 517 commitments (groups of stakeholders participating in the EIP on AHA) across all EU-countries (and beyond), which are organised in six thematic Action Groups (Table 1 and Figure 1) [5].

**Table 1:** Action Groups and number of commitments participating in the EIP on AHA.

Action Group	Action Group Theme	Participating Commitments
**A1**	Better prescription and adherence to medical plans for older patients	68

**A2**	Personalized health management, starting with a falls prevention Initiative	68

**A3**	Prevention and early diagnosis of frailty and functional decline, both physical and cognitive, in older people	131

**B3**	Replicating and tutoring integrated care for chronic diseases, including remote monitoring at regional level	125

**C2**	Development of interoperable independent living solutions, including guidelines for business models	59

**D4**	Innovation for age friendly buildings, cities and environments	66

Sources: [4,5]		

Focus area of each Action Group in the European Innovation Partnership on Active and Healthy Ageing, and number of commitments participating in each Action Group.

The monitoring framework developed comprises a web-based tool which rests on a Markov process and aims at estimating the impact of the EIP on AHA activities on health and on the sustainability of health and social care systems [6]. More precisely, the MAFEIP-tool allows estimating the change in quality adjusted life expectancy related to the activities carried out in the EIP on AHA and the estimated impact of a social or technological innovation on health and social care expenditure in a particular context [6].

This paper describes the approach taken for developing and testing the MAFEIP-tool, its main characteristics and the benefits it offers for estimating the health and economic outcomes of the activities carried out in the EIP on AHA. The following section describes how the main challenges for the monitoring of health and economic outcomes of the EIP on AHA were met, how the objectives of the EIP on AHA were operationalised and how this resulted in a short-list of indicators to assess the health and economic outcomes generated within the EIP on AHA commitments. We will further elaborate on the developed Markov model that initially rests on three generic health states ('baseline health', 'deteriorated health', and 'death') and which we aim to implement in a web-environment to allow for remote access and use by EIP on AHA stakeholders. Particular emphasis will be placed on the issue of variation across commitments which arises (amongst other things) from the diversity in terms of context, population, intervention and comparator, and also from differences in the evidence base and the methods used to collect outcome-related information. We also describe how a series of case studies covering different EIP on AHA Action Groups will be carried out in order to test our tool in the field. The cases selected all have a substantial eHealth, integrated care, or personalised healthcare component, and they will allow us to test different scenarios for the future rollout and further development of the tool: ranging from the pre-market assessment of early healthcare technologies to inform their further design, development and evaluation to the retrospective analysis of established care pathways based on administrative data and health records. In the discussion, we will further address the potential benefits of, and challenges for, the implementation and use of our tool for the systematic assessment of the social and technological innovations implemented in the context of the EIP on AHA, and we will also highlight potential areas for further development.

## Methods

### Conceptual framework

Probably the biggest challenge for developing a monitoring framework for the EIP on AHA is the contextual variation between commitments participating in the Partnership [6]. With more than 500 commitments spread across six thematic Action Groups which usually implement more than one social or technological innovation addressing various aspects of active and healthy ageing across Europe, immense variability needs to be accounted and controlled for [5-7]. The monitoring framework should therefore be general enough to be applicable across a large number of commitments but also sufficiently specific and sensitive to allow estimating the impact of a diverse set of interventions [6]. This trade-off has implications on all levels of the framework, starting from data collection on intervention level, the metrics chosen to express health and economic outcomes on partnership-level, the model to extrapolate from one level to the other, and the practical implementation of the model in form of a web-based tool [6-8]. The economic evaluation literature offers many examples of studies which have been adapted ex-post to different settings [e.g. [9-11]] and reviews by Sculpher et al. (2004) and Goeree et al (2007) show that decision analytic modelling (DAM) is a preferred means to adapt analyses to different contexts [12,13]. For this purpose, the method offers *'the maximum of consistency and the minimum of duplicative work*' [14] and it also allows to '*pull together the many needed pieces of information from multiple sources and to stitch them together into a (hopefully) cohesive whole' *[15]. Our approach to the monitoring of health and economic outcomes of the EIP on AHA rests on the same principles, though the number of different settings and the variation between them is probably unparalleled.

### Identifying suitable outcome indicators

The bottom-up approach of the EIP on AHA required identifying a short list of (mostly surrogate) indicators on intervention-level which a) are quantifiable, b) are able to capture the main effects of commitments across EIP on AHA Action Groups, and c) allow extrapolation towards a 'common currency', i.e. a generic measure of health impact which can be used to compare or aggregate outcomes across the different disease areas, interventions, and populations targeted in the Partnership. We choose incremental changes in Quality Adjusted Life Years (QALYs) of an intervention versus its respective standard care alternative in a particular context as common currency [16]. QALYs combine quantity of life (i.e. the length of life) and quality of life (i.e. the health states in which a certain length of life is being lived) into a single index, with one QALY being equal to one year in full health [16]. Following the common practice and on the basis of data availability, we identified measures of preference-based Health Related Quality of Life (HRQoL) and life expectancy derived from disease stratified mortality as preferred categories of health outcome on intervention level as they provide the information required for calculating QALYs. Preference-based HRQoL measures are particularly well suited for the purposes of MAFEIP as they can provide utility values which are:

• **defined on an interval scale **ranging from 0 (death) to 1(full health)

• **non-discriminatory**, so that an improvement from say 0.2 to 0.3 is valued identically to an increase from say 0.7 to 0.8, and

• **Additive**, so that the health benefits achieved across individuals, interventions and commitments delivered in the EIP on AHA can be aggregated

As one of the widest used HRQoL measures, the EQ-5D instrument is particularly well suited for our purposes as it is a generic, preference-based measure, with value sets and translations for most EU-countries (http://www.euroqol.org) [17]. It is also the method of choice to measure health benefits by a number of decision bodies internationally [18,19]. In addition, a survey on outcome indicators which we conducted across EIP on AHA stakeholders in early 2014 showed that the EQ-5D was the most commonly used instrument across the Partnership [7]. We observed, however, that commitments also reported collecting HRQoL with other instruments, such as the SF-36 [20], the SF-12, [21], the SF-6D [22], the Nottingham Health Profile [23], the 15D [24], or various disease specific instruments. In such cases, we consider 'mapping', or 'cross-walking' to convert the scores of other HRQoL instruments into respective EQ-5D values, whenever possible [25,26]. Different mapping strategies will be applied, depending on what level of data are available from commitments, whether mapping algorithms exist in the literature, and also depending on the extent to which the population samples used to estimate mapping algorithms match the samples reported by our commitments [27] The additional uncertainty related to the conversion of other HRQoL scores into their respective EQ-5D values will be examined in the context of parameter uncertainty analysis (see uncertainty analysis below). In addition to the above, we identified surrogate outcomes which are used either by some commitments across the Partnership or more regularly in a particular Action Group. These may potentially allow extrapolation towards more generic measures of health outcome, and amongst those surrogate indicators are measures of physical activity, risk factors (such as cholesterol levels, blood pressure, blood glucose or body mass index), adherence to treatment, falls, frailty, nutrition as well as functional status, mental health and cognitive decline [7].

To assess the economic outcomes of the interventions delivered in the EIP on AHA, we will estimate incremental changes in health and care utilisation of an intervention compared to its context specific standard care alternative, weighted with local unit-cost estimates [28]. This includes not only the cost of implementing and delivering one care alternative compared to another, but also the estimated impact on future disease related care utilisation [29]. We further consider relevant resource use outside the health sector, such as social care services, and also the cost falling on individual patients. The variety of EIP on AHA interventions which affect multiple budget holders (and potentially different budget holders in different jurisdictions) ultimately calls for a societal perspective [30,31], though the MAFEIP-tool will also allow switching between different economic perspectives.

### Model overview

At the core of the MAFEIP web-tool is a decision analytic model that integrates data from multiple sources to assess the impact of an intervention on for example life years gained, health-related quality of life and/or health- and social care costs. Decision analytic models have been widely used in economic evaluation of healthcare interventions as a framework for decision making under uncertainty regarding the true or "real world" effects and cost impacts of such an intervention[32]. Such models allow for the synthesis of evidence from multiple sources, the consideration of multiple comparators, the extrapolation of evidence over an appropriate time horizon, and account for the uncertainty associated with the decision[32]. Most decision analytic models are developed to assess a limited number of alternative interventions in a specific setting (i.e. disease, clinical setting and country). In such cases, design choices on the model structure and data input can reflect the specific purpose of the model, which will result in a model that is able to capture the differences in outcomes between alternatives with great detail. Contrarily, in the specific case of MAFEIP, the large variation of interventions to be analysed across multiple settings and populations requires an unusually high level of flexibility of the model.

### Model structure

**Figure 2:**
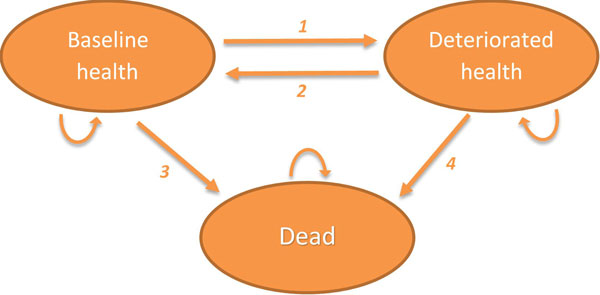
**Structure of the decision analytic Markov model**. (1) Probability to move from baseline health to deteriorated health. (2) Probability to move from deteriorated health to baseline health. (3) Probability to move from baseline health to dead state. (4) Probability to move from deteriorated health to dead state.

To achieve maximum flexibility, we base the tool on a Markov model with initially three mutually exclusive health states: 'baseline health', 'deteriorated health', and 'death' (Figure 2). Note that the optional inclusion of additional health states to further increase the flexibility of the tool is envisaged for further development, and we will pick up on this issue in the discussion. The baseline health state represents the HRQoL, life expectancy, as well as health and social care resource use (weighted with local unit cost) of the target population at which the intervention is aimed. For many interventions delivered within the EIP on AHA this will be the general population of an EU member state, or possibly a specific age group thereof [5]. The deteriorated health state represents a condition where one or more morbidities have negatively affected a person's HRQoL and life expectancy compared to the baseline health, and/or led to increased health and social care resource use. The specific definition of this state is determined by the nature and purpose of an intervention under assessment, as it represents the state of deteriorated health that the intervention aims to prevent or cure. Each state will have a HRQoL utility weight and two aggregate costs (healthcare and societal) attached to it. The simulated population will transfer between the Markov states based on four transition probabilities: the incidence of the deteriorated health condition (1), the rate of recovery from that back to baseline health (2), baseline mortality in the target population (3), and excess mortality in the population with deteriorated health (4) (numbers in parentheses refer to numbered arrows in Figure 2).

### Decision alternatives

The purpose of the MAFEIP-tool is to estimate the health and economic outcomes of a large variety of social and technological innovations in the health and care sector relative to current care. Each analysis therefore has two decision alternatives: the current care situation and the intervention as defined by the commitment. In the model, the two alternatives differ in terms of the transition probabilities (disease incidence, recovery and mortality), as well as the HRQoL weight, healthcare and societal costs attached to the health states. When the model simulates a hypothetical cohort of patients moving between these health states over time, the differences in survival, HRQoL and costs will accumulate to an estimate of the incremental costs (Δ*C*) and health effects (Δ*E*) that can be expected from the intervention under evaluation. The Commission does not intend to assess the incremental cost-effectiveness of an intervention carried out by an EIP on AHA commitment, nor to compare several interventions on their cost-effectiveness [6]. Rather, the general aim of the MAFEIP project is to estimate the aggregated impact of the EIP on AHA on its overall health and health system objectives [6]. However, the tool can be deployed by individual stakeholders to estimate the Incremental Cost Effectiveness Ratio $\left(\text{ICER}=\frac{\text{Δ}C}{\text{Δ}E}\right)$ or the Incremental Net Monetary Benefit (INMB = ΔE * λ - Δ*C*) of one intervention compared to another, where λ denotes the willingness to pay threshold for an additional unit of health gain [33].

### Parameter data

Multiple sources of evidence are needed to estimate the impact of an intervention. Each commitment in the EIP on AHA will generate evidence on the effects of their intervention(s), but this evidence will not always be sufficiently comprehensive or detailed to populate the entire model [6,7]. Evidence will then be complemented with literature data and expert opinion [15]. For those parameters that are not impacted by the intervention (i.e. mortality, HRQoL and resource use in the baseline health state) the tool will draw from a database that includes these data for each of the EU member states. While the default data set is derived from previously published data, users are encouraged to provide more specific (local) data to replace these parameter values and run the model with those values. Herewith the analyses become more relevant to their specific setting and likely more credible and actionable for policy makers [34]. In addition, all user-provided input data will be reviewed by the MAFEIP team. If deemed valid and credible, these data are stored in a separate database and made available to other users. Consequently, the tool will benefit from an ever expanding database of epidemiological, economic and effectiveness data that enables more accurate estimates of intervention impacts. Indeed, drawing from an ever increasing database with validated parameter estimates provided by, and of particular relevance for, members of the EIP on AHA community in their respective Action Groups is a key feature of the MAFEIP-tool, providing an added value to its users compared to existing decision models or commercially available decision modelling software.

### Uncertainty analysis

Uncertainty is inherent to any economic evaluation. The strength of decision analytic modelling is that the uncertainty and heterogeneity can be incorporated in the analysis, thus facilitating the assessment of its impact on outcomes [35]. Two types of uncertainty are present in decision analytic modelling: parameter uncertainty and structural uncertainty[30]. The latter stems from the assumptions that are made to capture a complex clinical process in a simplified model [36]. As the initial model for the MAFEIP-tool will retain the same structure for all analyses, structural uncertainty may only be relevant for subsequent versions of the tool and considered in more elaborate case studies conducted by the MAFEIP-team. Parameter uncertainty reflects the imprecise estimation of parameter values which are estimated for populations based on limited available information[32]. This imprecision will especially be large when the only evidence available for an intervention stems from a small pilot study, or some other form of early assessment, as opposed to more robust study designs, such as large randomized controlled trials.

How to incorporate uncertainty in the model outcomes depends on the quality of evidence available, i.e. the amount of uncertainty associated with parameter values. The current default strategy is to reflect parameter uncertainty in a probability distribution [32], which could be based on information available from commitments, on secondary data from the related literature, or even on expert opinion in cases no such information is available. This uncertainty is then propagated through the model by running it multiple times, each time with a different set of values drawn randomly from the probability distributions. This method, known as probabilistic sensitivity analysis, has the benefit that results fully reflect the uncertainty of the input parameters. However, for interventions that are not fully matured yet, in the sense that specific intervention characteristics (e.g. delivery mode of patient education, intensity of exercise training, specific content of a telemedicine interaction) are still to be determined, deterministic scenario analysis, where different sets of input parameters are varied consecutively, can be more informative to guide further development of the intervention[37]. As the MAFEIP-tool will be used for a wide variety of interventions in different stages of development, both probabilistic sensitivity analysis as well as deterministic scenario analysis will be incorporated in the tool. Users can determine themselves which analysis is most informative for them.

### Software

The decision analytic model is programmed in R, an open source statistical programming language [38]. Benefits of R for this application are its flexibility and computational efficiency. A drawback of R, however, is that it does not have a user interface by default, so that this needs to be developed specifically for the MAFEIP-tool.

### Web implementation

The ultimate goal of the MAFEIP-tool is to enable users who are not necessarily experts in the field of health economic modelling to perform an evaluation of their intervention with little to no third party support. Users will therefore interact with the model through a user friendly web-based interface. This interface will guide users through the steps that are required to perform an evaluation, and indicate the choices to be made and the options to select from. In the input forms, users will be able to select data from the database to populate the model, or provide their own data. Users can further control options and provide input via drop down menus, tick boxes and text fields. All input asked from the user will be accompanied with an explanation of the parameter, i.e. definition, theoretical value range (e.g. 0 to 1 for a probability parameter), and information on how user input is used in the model. Short descriptions will be provided directly in the interface, while more in-depth information will be provided via mouse-overs and expansion buttons. The user interface will be developed with PHP in the ZEND Framework, whereas its visible part (website) will be developed using HTML5 in combination with Javascript (jQuery / AngularJS) and will adhere to the Web Content Accessibility Guidelines 2.0 of the W3C. The data for calculations will be stored in MySQL and/or Couchbase (NoSQL based).

### Case studies and wider roll-out

Before making the tool accessible to a wider audience, we will conduct a series of case studies to test its applicability to different contexts, its user-friendliness, the need for third party support to populate parameters with adequate data and the added benefit it provides to users by estimating the health and economic outcomes of their respective interventions in the way described above. Cases will be selected so that they represent a wide variety of activities carried out within the EIP on AHA: we will therefore aim for cases from different Action Groups, implementing different social or technological innovations in different geographic and clinical contexts, aiming at different population subgroups and representing different implementation stages of their respective technologies; with the resulting variability in the available evidence base. Accordingly, cases will range from the pre-market assessment of early healthcare technologies to inform their further design, development and evaluation to the retrospective analysis of established care pathways based on administrative data and health records. What will be common to all cases, however, is their substantial eHealth, integrated care, or personalised healthcare component as this is also characteristic of the EIP on AHA as a whole.

Case studies will be carried out between February and September 2015 and upon their completion, a stakeholder workshop will be organised in order to gather the views from participants on their experience with the developed tool. This will then provide the basis for further improvements and for developing user-support strategies and materials which may be needed to facilitate the use of the tool by stakeholders with potentially limited experiences in health economic evaluation and decision analytic modelling. Public roll-out of the tool is envisaged end 2015.

## Discussion

The EIP on AHA brings together different stakeholders working, in the widest sense, on social and technological innovations to enhance active and healthy ageing, improve the sustainability of health and social care systems, and to generate innovation and growth opportunities for Europe [4]. Monitoring the activities that are carried out within the Partnership requires an approach that is highly flexible, whilst ensuring consistency in the way health and economic impact is estimated across interventions and commitments, and also feasible given the analytic expertise and resources available [6]. Our approach aims to address these challenges by developing a generic decision model, which rests on a Markov process implemented as a web-based tool with an expanding data base resulting from remote data input by EIP on AHA stakeholders. The sheer size of the EIP on AHA and the diversity of participating commitments provide compelling arguments in favour of this generic yet flexible approach, but there are two questions which inevitably follow: first, what is the value of the tool to users who generally aim for tailored analyses of their respective interventions in the context of their use in a particular setting, whilst the evidence available to populate parameters that drive the difference between decision alternatives is often very limited? Second, is it realistic to request that EIP on AHA stakeholders with potentially limited experience in health economic evaluation and decision analytic modelling execute unfamiliar tasks such as adapting the tool to a particular intervention and populating it with appropriate parameter estimates?

To answer the first question, it is important to keep in mind that the use of the described Markov model warrants our approach consistency, whilst the definition of three general health states ('baseline', 'deteriorated', 'dead') ensures flexibility to adapt the tool to different contexts. An important discussion, however, evolves around the appropriate number of health states provided by the tool. The generic three state model described here provides the minimum number of states to assess the impact of interventions that do not only aim at preventing or delaying death (in which case two states would be sufficient), but mainly at slowing down a deterioration in health, which is a typical characteristic of most interventions delivered within the EIP on AHA. The resulting tool allows adaptation to different target populations which vary in their respective definition of baseline and deteriorated health states, and this is ultimately reflected in population-, condition- and intervention specific transition probabilities, as well as local cost and effect valuations, whenever possible. However, the number of health states in a model should generally be driven by the research question, the nature of the disease and health technologies under assessment, and also the available evidence base. Hence, for a number of interventions delivered within the EIP on AHA, more than three health states may be appropriate. Providing the option to the user to include additional health states could therefore further increase the flexibility of the tool, which is why we consider this as a future development. Whilst automating this process in R and the tools' user-interface may require additional analytic resources but generally constitutes a solvable problem, potential limitations may relate to the availability of data to feed more complex models and the level of experience of the users of the MAFEIP tool. Due to the innovative character of many interventions delivered in the context of the EIP on AHA and the early stage of the Partnership as a whole, the availability of data to populate more complex models for estimating health and economic outcomes of an intervention may simply be questionable [6,7], though it is also likely that richer datasets to feed the MAFEIP tool will be accumulated over time.

Hence, a generic three-state Markov model that is flexible enough to deal with the contextual variation as described here, and which relies on relatively few but customizable input parameters (and their respective estimates of parameter uncertainty) may, at least for the early stages of MAFEIP, strike the best balance between the information needs on Partnership-level and the evidence base on intervention level, which is often limited and scattered [7]. The benefits of using the MAFEIP tool to provide initial estimates of expected effectiveness and costs of an intervention based on the best evidence that is currently available for a particular technology, and updating the model and its respective data inputs when further information becomes available in the future, are twofold: first, it provides users with an early assessment about the likelihood that their intervention in its current design will achieve the anticipated impact. Second, by identifying what drives the intervention's effectiveness or efficiency, the outcomes of the assessment can be used to guide further development of the intervention[39,40]. Such early evaluations are especially important in fields with rapid technological advancements, such as eHealth, personalised medicine, and integrated care to improve the efficiency of the innovation process[41].

This leaves us with the second question raised earlier: is it feasible to request from EIP on AHA stakeholders with potentially limited experience in the methods described here that they adapt and populate the tool with relevant data for their respective interventions? We believe that this implementation strategy is necessary as gathering all data by the MAFEIP team would simply not be manageable given the number of commitments, settings and interventions to be assessed within the EIP on AHA. However such an approach may only work if accompanied by a comprehensive on-going user-support strategy and it also poses another restriction on the complexity of the tool and its underlying model, particularly during the early stages of MAFEIP during which the diffusion of health economic evaluation concepts and methods within the EIP on AHA community is likely to be low. The tool interface, which aims to guide users intuitively through the process of adapting and populating the model with data, and provides a wealth of background information, including reference to, and extensive discussion of, existing good practice guidelines for state transition modelling [42], can only serve as a starting point. We believe that further support will be needed to ensure efficient implementation of the MAFEIP-tool, and we intent to facilitate this support through the channels provided by the EIP on AHA. Indeed, we believe that the Partnership provides an excellent platform to facilitate our approach, to organise workshops and seminars on the topic and to train future 'MAFEIP-advocates' within their respective Action Groups who will then contribute to further implementation. There are examples in the literature, both for developing web-based Markov tools similar to the one we propose for the purposes of MAFEIP (e.g.: http://www.nottingham.ac.uk/match/research/tools/markovtoolmain.html), and also for facilitating the approach to an audience with little or no background in economic evaluation and decision modelling methods [43,44]. In this context, Crowe et al. (2010) state that *'even though small companies and healthcare purchasers have little prior knowledge of health economics, the key issues can be rapidly absorbed to be applied in decision making*.'[43] Ultimately, our planned implementation strategy may also foster the diffusion of health economic evaluation principles and methods in the decision making process in eHealth, personalised medicine and integrated care, which will hopefully be perceived as another benefit of this project.

## Conclusion

Monitoring the activities carried out within the EIP on AHA requires an approach that is both flexible and consistent in the way health and economic impact is estimated across interventions and commitments. Our approach aims to address these challenges by developing a generic decision model, which rests on a Markov process implemented as a web-based tool with an expanding data base resulting from remote data input by EIP on AHA stakeholders. The added value for users of the tool will be its ability to provide an early assessment of the likelihood that interventions in their current design will achieve the anticipated impact, and also to identify what drives interventions' effectiveness or efficiency to guide further design, development or evaluation. Public rollout of the tool should be accompanied by a comprehensive user-support strategy provided through the channels of the EIP on AHA, which may also contribute to the wider diffusion of economic evaluation methods in key areas of the EIP on AHA, including eHealth, personalised medicine and integrated care.

## Competing interests

The authors declare that they have no competing interests.

## Authors' contributions

CEHB is the principal investigator of the MAFEIP research project. He developed the design for this study and tools for data collection, undertook data analysis, drafted the first manuscript and coordinated its revision. GdG and LS are responsible for programming the monitoring tool and its web-implementation, and they are also involved in case study development and execution. Both contributed to drafting and revising the manuscript, in particular sections related to model development and implementation. YY is an NIHR (Oxford Biomedical Research Centre) funded researcher who served as a consultant for this project on topics related to HRQoL measurement and mapping, and she also contributed to drafting and revising respective sections in the manuscript. FA is managing the MAFEIP project and contributed to drafting and revising the manuscript. All authors read and approved the final manuscript.

## Disclaimer

The views expressed are purely those of the authors and may not in any circumstances be regarded as stating an official position of the European Commission. The Commission is not responsible for any use that may be made of the information the article contains.

## Declarations

This research was developed under the Monitoring and Assessment Framework for the European Innovation Partnership on Active and Healthy Ageing (MAFEIP) project funded jointly by the European Commissions' Joint Research Centre, Institute for Prospective Technology Studies (JRC IPTS) and European Commission Directorate General for Communications Networks, Content & Technology (DG CONNECT). MAFEIP was launched in September 2012 and is due to be completed in November 2015. Grant number 32846-2012-08. Publication cost were funded by the Joint Research Centre.

This article has been published as part of *BMC Medical Informatics and Decision Making *Volume 15 Supplement 3, 2015: Multidimensional, multidiscipline and shared decision making in healthcare and eHealth. The full contents of the supplement are available online at http://www.biomedcentral.com/bmcmedinformdecismak/supplements/15/S3.
